# Occupational Health Risk of Waste Pickers: A Case Study of Northern Region of South Africa

**DOI:** 10.1155/2021/5530064

**Published:** 2021-08-30

**Authors:** Solomon E. Uhunamure, Joshua N. Edokpayi, Karabo Shale

**Affiliations:** ^1^Department of Environmental and Occupational Studies, Faculty of Applied Sciences, Cape Peninsula University of Technology, Cape Town 8000, South Africa; ^2^School of Environmental Sciences, University of Venda, Thohoyandou X5050, South Africa

## Abstract

In South Africa, waste pickers play a significant role in the management of waste at landfill sites. Waste picking is an income-generating venture for most people with low-income base. The activity of sorting waste at landfill sites is, however, associated with occupational health risks to waste pickers which this study has examined. The study adopted a cross-sectional survey with a convenience sampling method which was conducted among 114 waste pickers in three landfill sites in Limpopo Province of South Africa. A validated questionnaire was used in eliciting responses from the participants. The statistical technique employed includes the ANOVA, simple, and multiple regression. The results indicated that, in the last one year, waste pickers exposed to landfill sites were 1.7 times more likely to develop a common health disorder (AOR: 1.733; 95% CI: 1.069, 2.755; *P* value: 0.041). There was statistical significance between the number of days worked at the landfill and the health conditions of the waste pickers (*P* ≤ 0.001). The cofounders were adjusted for age and years worked, and the result revealed that days worked by the waste pickers' increased their chances of occupational health risks by 1.4 times. It is unlikely that waste pickers will have a risk-free environment, but supportive policies such as provision of adequate personal protective equipment and more awareness programmes on the health risks related to such enterprises will aid in abating the associated risks.

## 1. Introduction

Globally, the generation of solid waste has increased tremendously over the last decade. Hazardous and toxic materials, recyclables, and other useful materials are usually components of the waste stream [1, 2]. According to the World Bank, waste generation has intensified to the point where it will double by the year 2025; thus, there is indispensable need for improvement practices of solid waste management [3, 4]. Worldwide, around 1.3 billion tonnes of waste are generated annually, and it is anticipated that 2.2 billion tonnes of waste will be generated by 2025. In variance with the sophisticated approaches employed by developed countries in waste management strategies, many developing countries are still struggling with the disposal of waste generated [5]. In contrast to the developing countries, most developed countries have implemented and adhere to strict waste classification and separation system which is a critical link in the recycling system. The classification system helps in ensuring that most recyclable resources are separated from household waste and which significantly simplifies waste disposal [6].

In South Africa, 54,425 tonnes of waste are generated daily, placing the country as the 15th highest waste producers in the world [4].

According to the Department of Environmental Affairs (DEA), in 2011, approximately 10% of South Africa's waste was recycled. The balance 98 million tonnes of unrecycled waste ended in landfill sites [7]. A significant but least recognised component in promoting recycling are the efforts of estimated 90,000 waste pickers in South Africa, who earn their living mainly from recyclables and reusables, either from landfill sites or on the street [8]. Over the years, waste pickers have played a pivotal role in managing waste through the recycling process, although in the recycling value chain, waste picking activities are at the lower end [9]. Waste pickers are commonly referred to as scavengers, waste scavengers, reclaimers, or garbage pickers [10]. Often, waste pickers are self-employed, small-scale agents, typically found in the informal urban sector [11]. Rapid urbanisation coupled with a high unemployment rate of 30.8% in South Africa is one factor leading to a rise in informal sector activities such as waste picking [12]. In most developing countries like South Africa, uncontrolled urbanisation leads to an upsurge in urban poverty and inequality, thus creating avenues for more people to be involved in more informal activities [13]. More so, globally, sub-Saharan Africa is regarded as one of the fastest urbanising regions as well as exhibiting high poverty tendencies.

South Africa adopted four stages of economic instruments in mapping the waste legislative context over the past three decades through which the waste and recycling sector has transitioned. The first is known as the “The Age of Landfilling” which started around 1989. The second stage is the “Emergence of Recycling” which started around 2001, leading to the banning of single-use plastic bags. The second saw the growth of waste economy in the country; however, only about 10% of the generated waste were diverted from the landfill towards recycling. The third stage, tagged “The Flood of Regulation” started in 2008 with the promulgation of the National Environmental Management Waste Act (Act 59 of 2008) [14]. This resulted in a wave of new guidelines aimed at regulating the waste and secondary resources sector. The fourth stage, known as “The Drive for Extended Producer Responsibility started around 2012, managed by the Producer Responsibility Organisation was envisioned in fulfilling the producers” responsibilities for end-of-life waste through a compulsory Extended Producer Responsibility scheme [15].

Cooperatives have been actively promoted by the government as a way of formalising the informal sector, ensuring enterprise development and stimulating job creation. Nonetheless, at 91.8%, waste and recycling cooperatives have enmeshed in high failure rate [16]. Most of the cooperatives face several challenges which include premises to sort and store recyclables and lack of access to transport and equipment. More so, operational and capability challenges such as theft of recyclables, difficulties in accessing market, and weak competence to operate a business were found to hinder the successful implementation of these cooperatives [17, 18]. Copious initiatives and research developments are ongoing in the country to find seemly resolutions to incorporate the informal waste sector into the local waste economy as there is still much to do in strengthening the local recycling economy. The waste sector has been acknowledged in the national strategy and policy documents as a sector that can contribute towards the economic growth and creation of green jobs in the country [19, 20].

Waste pickers are often marginalised individuals engaged in picking and sorting of waste for sale, but doing so under a high-risk exposure of occupational and environmental hazards [21]. Potentially present in landfill sites are several chemicals known to have detrimental effects on human health [22–24]. Typically, the waste which is accrued in a landfill site is commoditized and created further as reused or recycled goods. Waste pickers build their livelihood around resource salvaging and are mostly unaided and short of adequate health protection measures in place [25]. The understanding that waste picking can pose a serious health risk to human health and the environment is well documented [26]. Associated with landfill sites exposure are health problems such as dermatological symptoms, headache, gastrointestinal symptoms, mental illnesses, chronic and infectious diseases, and allergies among others [27].

Undoubtedly, most landfill sites are faced with inadequate health and safety measures, which include the use of personal protective equipment (PPE) and hazard awareness that could negatively impact the health status of the waste pickers. Hence, the health conditions of the waste pickers could be adversely affected. Due to cuts and contact with toxic substances like dust inhalation, bacteria, and chemicals, a large proportion of waste pickers are at a higher risk of being infected by injuries and diseases unlike the general population [28, 29]. Health impact of waste pickers is also affected by their socioeconomic characteristics, such as age, educational level, income earned, and number of days worked weekly [25, 30–34]. An overlooked but critical research area in South Africa is the associated health risks faced by waste pickers [35–38]. The objective of this study, therefore, is to assess the dynamics of occupational health risk of waste pickers in South Africa, using three landfill sites in the Limpopo Province. The study hypothesis is aimed at establishing the epidemiological evidence of the potential health impact associated with waste picking at landfill sites. The considered variables among others include health-related symptoms, mental health, use of personal protective equipment, and infectious and chronic diseases. The study results would provide information that can serve as a basic impetus for stakeholders and for further research on the health impact of waste pickers in landfill sites.

## 2. Materials and Methods

### 2.1. Description of the Study Area

**Figure 1:**
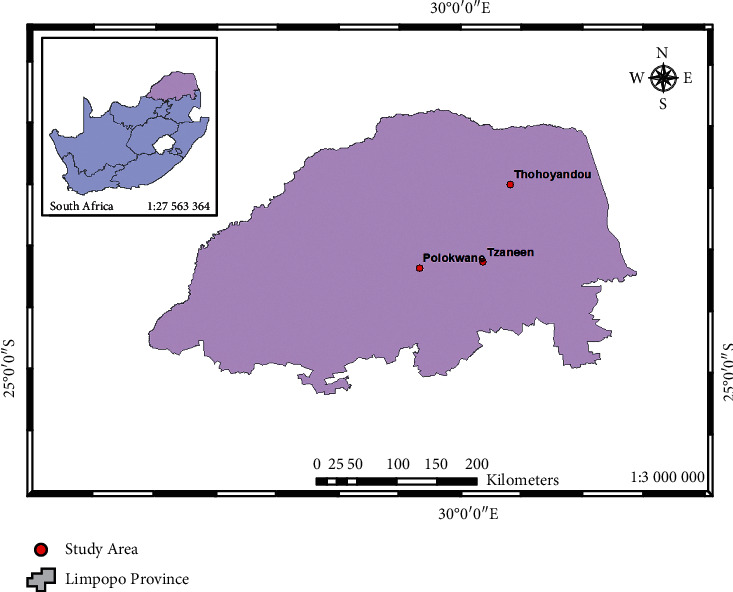
Map of South Africa showing the study areas.

Figure 1 shows the map of South Africa indicating the location of the study areas. The province lies between coordinates 23° 40ʹ 13.81ʺS and 29° 41ʹ 79.90ʺE. Limpopo Province is the northernmost province of South Africa, lying within the curves of the great Limpopo River [39]. Characterised in the province is an unequal access to basic amenities and resource distribution. Due to the high unemployment level, most of the households depend on governmental grants and allowances from household members who migrate to work in other provinces [40].

In South Africa, landfilling of hazardous and general waste remains the dominate technology solution, with approximately 90% of all waste generated in the country are disposed in landfill [41]. Typically, this is the adopted method of waste management in most developing countries, where wastes are disposed in landfills [42]. The landfill sites in the study areas have parameter fencing, with weighing bridge at the gate except for Thohoyandou landfill. Usually, high volumes of waste are received daily. These wastes consist of garden waste; domestic waste; commercial, industrial, and business waste; and building rubble. The composition of these incoming wastes to the landfill sites differs according to the generation of the different waste as well as the environmental and social conditions and the entities operating the sites. Polokwane landfill site receives higher volume of waste than Tzaneen and Thohoyandou landfill sites. These landfill sites are licensed to receive hazardous waste. Clay bentonite is used as liners to prevent leachate from sipping underground.

## 3. Data Collection and Sampling Method

This study adopts a cross-sectional survey design conducted at three landfill sites of the Limpopo Province. The exposed subjects (waste pickers) were proportionally drawn from the three largest landfill sites in the province. Due to differences in landfill sizes and number of waste pickers, 48, 16, and 50 participants were drawn from the Tzaneen, Thohoyandou, and Polokwane landfill sites, making a total of 114 participants. The landfills are henceforth represented as A, B, and C for Tzaneen, Thohoyandou, and Polokwane, respectively.

This study employed a statistical equation in calculating the sample size from the target population. The sample size was obtained based on three factors: (i) the desired confidence level, (ii) the assumed proportion of the sample, and (iii) margin of error. The Cochran equation was adopted in ascertaining the sample size, which according to Godden [43] is stated as

(1) $$\begin{array}{c} n=\frac{Z\left[{}^{2}p\left(1-p\right)\right)}{m^{2}}, \end{array}$$

where *n* = sample size. *Z* = *z* value (1.96 at 95% confidence level). *p* = proportion of the estimated sample. *M* = margin of error (assumed at 0.05).

(2) $$\begin{array}{c} n=\;\frac{{\left(1.96\right)}^{2}\times 0.05\times \left(1-0.05\right)}{{\left(0.05\right)}^{2}}.\\ \text{Hence,}\\ =114. \end{array}$$

Participation in the study was solely voluntary, and an informed consent form was signed prior to data collection. The waste pickers at the three landfill sites are volunteers. However, they have to be registered with the landfill management before access is granted to them to engage in any activity as deemed necessary by the landfill management. To ensure quality assurance and quality control of the data to be collected, the questionnaires were pretested before the main survey with adjustments and corrections made based on the responses to improve clarity. The questionnaires used in eliciting data from the respondents were self-administered, semistructured, and divided into four sections. Section A entails demographic and socioeconomic characteristics information including gender, age, marital status, educational level, income earned, and the number of dependants. Section B includes the types of waste sorted and the use of PPE. Section C entails questions dealing with associated health symptoms in the last year which include respiratory, gastrointestinal, dermatological, musculoskeletal, eye, and ear symptoms. Section D dwells on self-rated health conditions such as mental health, use of alcohol, smoking habit, and landfill safety. For convenience and simplicity, the questionnaires were administered in English language and where indispensable interpreters were used in local dialects. Trained research assistants from the University of Venda were engaged to execute and oversee the administration of the questionnaires, which was thoroughly monitored for data quality assurance.

## 4. Data Analysis

Data cleansing and statistical analysis were carried out using Statistical Package for the Social Sciences (SPSS) version 24 developed by International Business Machine (Armonk, NY, USA). Descriptive statistics were applied in analysing the sociodemographics of the respondents captured data. The analysis of variance (ANOVA) was used to compare the association difference between two or more independent groups, while differences in numerical variables were determined using the *t*-test. Logistic regression analysis was employed to determine the association between waste picking and health status of the respondents. The maximum likelihood ratio was used in ascertaining the model goodness-of-fit, describing the association between landfill exposure and the independent variables such as gender, age, educational level, income, and associated health symptoms. The method of validating the goodness-of-fit of the regression model is the Hosmer and Lemeshow test (ꭕ^2^ test), and it is considered more robust, particularly if the samples are small. The presented results were in crude and adjusted odds ratios, confidence interval (95%). The level of statistical significance of 0.05 was considered.

## 5. Ethical Clearance

This study was approved by the Ethical committee of the University of Venda (certificate number: SES/17/HWR/03/1510), to ascertain the avoidance of harm to the participants, and informed consent of the participants were obtained prior to the commencement of the study. Necessary permissions were obtained from the landfill management and appropriate government authorities.

## 6. Results

The sociodemographic characteristics of the respondents as presented in Table 1 indicated that there were 60 female and 54 male waste pickers across the three landfill sites. A majority of the waste pickers were within the age group of 31–40 years, representing 53% of the total sample. The results regarding age also indicated that there were no child waste pickers recorded at the landfill sites, mainly because the landfill sites were licenced and underage waste pickers are not allowed to be engaged in waste picking activities. The marital status indicated that 30 (62.5%) of the respondents in landfill A were married, 7 (43.8%) in landfill B, and 20 (40%) in landfill C. Landfill A recorded 19 (39.6%) respondents as attaining secondary education, 9 (56.3) were recorded in landfill B, and 29 (58%) in landfill C. Monthly income earned by the waste pickers indicated that, in landfill A, 22 (45.8%) of the respondents earned between R1001–1500. Landfill B indicated that 10 (62.5%) of the respondents earned between R501 and 1000. In landfill C, 29 (58%) of the respondents earned between R1001 and 1500. The number of years worked as waste pickers shows that 46.5% have worked between 1 and 3 years; 24.6%, between 4 and 5 years; and 28.9%, 6 years and above. The reported number of days worked weekly indicates that 36% of the respondents worked all days of the week, 34.2% worked for 6 days weekly, 26.3% worked 5 days per week, 1.8% worked 4 days a week, 0.9% worked 2 days, none for 1 day weekly work, and 0.9% did not tell.

The association between waste picking and health symptoms in the last one year was statistically tested as indicated in Table 2. The analysis of variance (ANOVA) result indicates a significant relationship between waste pickers and infectious and chronic diseases. Across the three landfill sites, 11.4% rated their health condition as very good, 49.12% as good, 31.58% as average/fair, and 5.26% rated their health status as poor. The response to the mental health condition of the waste pickers shows that 78.95% indicated that they are not at risk as against 21.05% who agreed to be at risk. Clinic/hospital visit in the last year showed that 54.39% had visited the clinic/hospital for consultation and treatment as against 45.61% who never visited the clinic/hospital.

There was no statistical significance between waste picking and mental health disorder (*P* value 0.460) as indicated in Table 2. However, with adjusted cofounders between landfill exposure and mental health disorder, as shown in Table 3, the result of the multiple logistic regression analysis conducted indicated in the last one year that waste pickers exposed to landfill sites were 1.5 times likely to develop a mental health disorder (AOR: 1.540; 95% CI: 1.252, 5.664; *P* value: 0.003).

There was statistical significance between the numbers of days worked at the landfill and the health conditions of the waste pickers (*P* ≤ 0.001). The cofounders were adjusted for number of days worked as shown in Table 4, and the results revealed that days worked by the waste pickers' increased their chances of occupational health risks by 1.4 times due to landfill exposure.

Results in Table 5 show that, for the past 1 year, there is no statistical difference between waste picking and infectious diseases (*P* value: 0.261) and between waste picking and chronic diseases (*P* value: 0.518). Nonetheless, using the adjusted cofounders, the multiple logistic regression indicated a statistical significance between waste picking and infectious diseases (AOR: 2.081; 95% CI: 1.349, 2.109; *P* value: 0.021) and chronic disease (AOR: 2.136; 95% CI: 1.406, 2.254; *P* value: 0.004). The adjusted crude ratio indicates waste pickers are 2 times more likely to have infectious and chronic diseases.

Table 6 indicates the occupational and hazardous exposures encountered by waste pickers in the landfill sites. Common hazardous substances and dust from building rubbles and other waste materials were observed as the main sources of exposure.

## 7. Discussion

In South Africa and like in most developing countries, the adverse health risks faced by waste pickers have not received much attention [35]. The occupational health risks faced by waste pickers require attention from relevant stakeholders. The objectives of this study were to investigate the occupation and environmental health risk associated with waste picking from the perspective of health symptoms, self-rated questions, and clinic/hospital visits. The study found that waste pickers are significantly at a higher risk of occupational health risk. Supported by the findings from the study are several related community health surveys that investigate a wide range of health complications related to environmental exposure to a landfill [44]. Increased occurrences of associated symptoms between waste picking and landfill such as illnesses, self-rated health, and clinic visit have been reported [45]. Reported study has also revealed the relationship between waste picking and mental health disorders [24]. Associated health problems such as musculoskeletal, gastrointestinal, dermatological, eye, and ear symptoms faced by waste pickers have also been reported [46].

Although, the result from this study indicates that smoking is not significantly associated with occupational health symptoms faced by waste pickers. The study result, however, reported that, from the three landfill sites, 50% of respondents engaged in smoking. Studies have documented that the prevalence of respiratory symptoms has a significant association with smoking. Hence, the prevalence of respiratory symptoms increases with smoking [47]. Studies have also shown that there is a higher occurrence of smoking within low socioeconomic groups, particularly amongst deprived individuals such as waste pickers with negative emotions as a result of coercion and humiliation encountered [48]. Hence, as a coping mechanism to help alleviate emotional trauma, some may take up smoking [49]. The study results portrayed the odds that those with a history of smoking are more likely to report respiratory symptoms. Smoking is a known confounder to respiratory symptoms with occupational exposure and negative effects [47].

Work conditions and practices observed among the three landfill sites were different. Although virtually all waste pickers in the landfill sites concur to using PPE while working, the observation shows that the waste pickers were not properly kitted with the recommended PPE, such as the absence of eye goggle and the recommended use of the N95 or dust masks. The recommended masks can protect the infiltration of airborne dust from the landfill sites which can penetrate through their respiratory system. The use of appropriate and recommended PPE are essential components in safeguarding their occupational health. Often, waste pickers collect worn or old shoes and clothes from landfill sites and use it as PPE [45]. Health impact of waste pickers may be susceptibly enhanced by inappropriate use of PPE [30]. Interrelated studies have found that most waste pickers complained of respiratory symptoms from landfill sites [50]. There are concerns of visible dust which are a common occurrence in landfill sites, mostly when garbage trucks are offloading their waste, thereby exposing them to occupational health risks. Waste pickers in the study area search through the waste for cardboards, cans, wooden materials, and bricks, among others. These collections often increase the amount of dust which can irritate the mucous membrane and the respiratory tract [51]. To further avoid occupational health risks related to dust, there is an urgent need for proper PPE to be worn among the waste pickers.

The result of our findings reveals a significant association between waste picking and dermatological and gastrointestinal symptoms. This is in line with a cross-sectional study conducted among waste pickers in Kota Bharu, Malaysia, which reported that 70.3% had dermatological symptoms and 65.5% had gastrointestinal symptoms [46]. In the study area, due to the high burden of illnesses associated with waste picking, 54% of the waste pickers reported that they have visited the clinic/hospital in the last 1 year. The result differs from that obtained in Brazil by Auler et al. [52] which reported 63% clinic/hospital visit from a sample size of 1000 participants. The study results also contradict the findings of Made et al. [44], in a study conducted in Johannesburg, where 41% of the respondents indicated visiting the clinic/hospital in the last 1 year which was based on 361 respondents. The result also varies from the findings in Colombia, which was based on 174 respondents. According to Gomez-Correa et al. [53], 32% of the waste pickers reported to be visiting the clinic/hospital in the last year.

The study result found that, about 50% of the respondents rated their health condition as good. The finding can be related to a study which reported that 43% of the waste pickers surveyed in Johannesburg reported their health status as good [45]. Conversely, another study has reported more than 50% of the participants indicating their health condition as average [54]. This may be attributed to the fact that waste pickers in the Limpopo Province do not consider working in landfill sites as an occupational hazard since the need for money takes priority over their health. However, studies have also revealed that due to possible eviction fears from work, waste pickers are not likely to report their health status as poor. This perhaps could also be an influencing factor amongst the waste pickers in the three sampled landfill sites [45, 55].

The results of musculoskeletal symptoms revealed that there is a statistical significance between waste picking and body pain in the three landfill sites. In landfill A, 34% of the respondents indicated that they lift heavy objects while rummaging waste. In landfill B, the reported number was 45%, and in landfill C, 23% recounted lifting heavy objects. Waste picking entails frequent kneeling which occurs when waste is being sorted and collected, which is linked to lower-extremity pain. Moreover, heavy lifting causes back and shoulder pain, tendon diseases, increased pulmonary ventilation, and lumbar disc prolapse [56]. The result of this study differs from the results reported by Aminuddin and Rahman [46] in Kota Bharu, Malaysia, where about 94% of the respondents indicated musculoskeletal symptoms from waste picking.

The use of protective goggles is one possible measure to reduce health risks, particularly for waste pickers working in a landfill site. Eye protection helps prevent infectious diseases which can emanate from the landfill and also cuts and injuries that can specifically affect the eyesight from sharp materials such as broken bottles and glasses [57]. Results and observations from the three landfill sites indicated that none of the waste pickers use eye goggle. However, access to eye goggle as a PPE is rare with waste picking activities, and where available, waste pickers do not always wear them [58]. The odds of occupational health risks are prevalent with waste pickers that have a history of reported common infectious diseases as with those that do not [45]. According to Thakur et al. [59], in a longitudinal study conducted in India, waste pickers with poor self-rated health were three times more likely in developing infectious diseases. Waste pickers are always in direct contact with toxic materials in waste at the landfill sites [25]. Infections from chemical substances, heavy metal, and other dangerous wastes put waste pickers at adverse risk of developing occupational health hazards, and this is worsened by using inadequate and improper PPE [45]. The findings from the study using ANOVA, however, indicated there was no significant association between infectious diseases and the occupational health of the waste pickers. Among waste pickers with adjusted cofounders, respondents with a history of chronic diseases were two times more likely to report their health status as poor when compared to respondents without history of chronic diseases. Association between chronic diseases and poor self-rated health was reported by Machado et al. [60]. A study by Bacok et al. [61] in Malaysia reported a significant relationship between chronic diseases such as hypertension and diabetics and landfill exposure. In addition, a study in South Africa revealed a statistical significance between landfill exposure and health impact. The identified impacts include dust, eye irritation, and skin disorders [62]. The result of the study indicates a statistical significance between landfill exposure of waste pickers and the chances of chronic diseases.

## 8. Limitations

This study offers a preliminary synopsis of the occupational health risks faced by waste pickers in the Limpopo Province, but the results cannot be generalized for the country as only three landfill sites in the province were selected. The results indicate more sensitive indicators of risk exposure by waste pickers. Epidemiological study of this nature requires a larger sample size of thousand participants. More so, as a cross-sectional study, a convenient sampling method was employed, thus could not accurately denote the entire community of waste pickers. Additionally, due to fear of apparent repercussions from landfill management, waste pickers could also present bias information as they may not be truthful in responding to some questions during data collection. Relied upon by the study were self-rated questions on disease history which may recall bias answers by the participants as a result of social desirability, as some may over-report their health conditions in order to be viewed as good by the data collectors.

## 9. Policy Implications

The health status of waste pickers is largely influenced by the activities on the landfill sites. Significant associations were observed between landfill exposure and mental health disorders and infectious and chronic diseases. Implications drawn from the study revealed that waste pickers are at a risk of occupational health hazards, and it is hoped that the results will attract the attention of the relevant stakeholders. From the local to national levels, there is a need to continuously monitor the health and socioeconomic conditions of the waste picker by designing and implementing risk prevention programs from generated baseline information and reliable statistics. More awareness programmes that could help waste pickers identify key health symptoms at an early stage are encouraged. Training on the handling of hazardous substances at the landfill site is also needed. Provision of adequate PPE and proper hygiene practices could reduce the burden of illnesses faced by waste pickers when rummaging waste. In addition, the provision of a mobile clinic at the landfill can serve as the first point of care for the waste pickers. Above all, a good waste governance at all tiers of government is advocated.

## 10. Conclusion

This study provides epidemiological evidence faced by waste pickers at landfill sites and serves as a yardstick for future research on the impact of a landfill in South Africa. There was no statistical significance between waste picking and mental health disorder (*P* value: 0.460); however, with adjusted cofounders, the result of the multiple logistic regression analysis conducted indicated that, in the last one year, waste pickers exposed to landfill sites were 1.5 times likely to develop a common health disorder. The results further indicated significant associations between occupational health of the waste pickers and landfill exposure, number of days worked and age, and infectious and chronic diseases. The study results have implications on the waste pickers due to the occupational and hazardous exposures in the landfill sites. It is hoped that this research will serve as impetus to stakeholders, districts, municipalities and provincial health officers in mitigating the health status of the waste pickers. Furthermore, it is suggested that future research on a larger scale that will include landfills from other provinces should be considered. A cohort study in monitoring and evaluating the health status of the waste pickers will offer a resilient epidemiological path of the adverse effects of landfill sites. Additionally, risk management and environmental monitoring will serve as a yardstick for the scientific basis in measuring the health effects of the waste pickers.

## Figures and Tables

**Table 1:** Socioeconomic demography of waste pickers.

Demography and socioeconomic characteristics	Total frequency (%)	Landfill A *n* (%)	Landfill B *n* (%)	Landfill C *n* (%)	*P* value
*Gender*					
Male	54 (47.4)	18 (37.5)	5 (31.3)	31 (62)	0.019
Female	60 (52.6)	30 (62.5)	11 (68.8)	19 (38)	
Total	114 (100)	48 (100)	16 (100)	50 (100)	
*Age (years in bracket)*					
12–20	—	—	—	—	≤0.001
21–30	15 (13.3)	5 9 (10.4)	—	10 (20)	
31–40	60 (53.1)	24 (50)	7 (43.8)	29 (58)	
41–50	33 (29.2)	19 (39.6)	3 (18.8)	11 (22)	
51 and above	5 (4.4)	—	5 (31.3)	—	
Did not tell	1 (0.9)	—	1 (6.3)	—	
Total	114 (100)	48 (100)	16 (100)	50 (100)	
*Marital status*					
Single	38 (33.3)	8 (16.7)	6 (37.5)	24 (48)	0.035
Married	57 (50)	30 (62.5)	7 (43.8)	20 (40)	
Divorced	10 (8.8)	7 (14.6)	—	3 (6)	
Widow/widower	7 (6.1)	3 (6.3)	1 (6.3)	3 (6)	
Did not tell	2 (1.8)	—	2 (12.5)	—	
Total	114 (100)	48 (100)	16 (100)	50 (100)	
*Highest level of education*					
No formal education	1 (0.9)	—	1 (6.3)	—	0.153
Primary	55 (48.2)	29 (60.4)	5 (31.3)	21 (42)	
Secondary	57 (50)	19 (39.6)	9 (56.3)	29 (58)	
Basic degree	1 (0.9)	—	1 (6.3)	—	
Honours degree and above	—	—	—	—	
Total	114 (100)	48 (100)	16 (100)	50 (100)	
*Years of employment*					
1–3	53 (46.5)	29 (60.4)	1 (6.3)	23 (46)	≤0.001
4–5	28 (24.6)	12 (25)	4 (25)	12 (24)	
≥6	33 (28.9)	7 (14.6)	11 (68.8)	15 (30)	
Total	114 (100)	48 (100)	16 (100)	50 (100)	
*Monthly income (Rand)∗*					
0–500	1 (0.9)	—	1 (6.3)	—	
501–1000	37 (32.5)	13 (27.1)	10 (62.5)	14 (28)	0.052
1001–1500	54 (47.4)	22 (45.8)	3 (18.8)	29 (58)	
3501–5000	4 (3.5)	2 (4.2)	2 (12.5)	—	
≥5001	1 (0.9)	1 (2.1)	—	—	
Total	114 (100)	48 (100)	16 (100)	50 (100)	
*Working days*					
1	—	—		—	0.004
2	1 (0.9)	1 (2.1)		—	
3		—		—	
4	2 (1.8)	—	2 (12.5)	—	
5	30 (26.3)	11 (22.9)	7 (43.8)	12 (24)	
6	39 (34.2)	19 (39.6)	5 (31.3)	15 (30)	
7	41 (36)	17 (35.4)	1 (6.3)	23 (46)	
Did not tell	1 (0.9)	—	1 (6.3)	—	
Total		48 (100)	16 (100)	50 (100)	

∗1USD equals R 15.6.

**Table 2:** Waste picking and associated health symptoms in the last 1 year.

Health symptoms	Total frequency (%)	Landfill A *n* (%)	Landfill B *n* (%)	Landfill C *n* (%)	*P* value
*Respiratory symptoms*					
Yes	14 (14.28)	—	7 (43.75)	16 (32)	0.006
No	73 (64.04)	48 (100)	6 (37.5)	19 (38)	
Did not tell	27 (23.68)	—	3 (18.75)	15 (30)	
Total	114 (100)	48 (100)	16 (100)	50 (100)	
*Gastrointestinal symptoms*					
Yes	8 (7.02)	—	6 (37.5)	5 (10)	0.002
No	64 (56.14)	47 (97.92)	3 (18.75)	16 (32)	
Did not tell	42 (36.84)	1 (2.08)	7 (43.75)	29 (58)	
Total	114 (100)	48 (100)	16 (100)	50 (100)	
*Dermatological (skin) symptoms*					
Yes	12 (10.53)	8 (16.67)	7 (43.75)	11 (22)	≤0.001
No	60 (52.63)	40 (83.33)	3 (18.75)	17 (34)	
Did not tell	42 (36.84)	—	6 (37.5)	22 (44)	
Total	114 (100)	48 (100)	16 (100)	50 (100)	
*Musculoskeletal symptoms*					
Yes	16 (14.04)	—	10 (62.5)	12 (24)	≤0.001
No	60 (52.63)	38 (79.17)	2 (12.5)	19 (38)	
Did not tell	38 (33.33)	10 (20.83)	4 (25)	19 (38)	
Total	114 (100)	48 (100)	16 (100)	50 (100)	
*Eye symptoms*					
Yes	14 (12.28)	11 (22.92)	8 (50)	6 (12)	≤0.001
No	41 (35.96)	32 (66.67)	2 (12.5)	4 (8)	
Did not tell	60 (52.63)	5 (10.42)	6 (37.5)	40 (80)	
Total	114 (100)	48 (100)	16 (100)	50 (100)	
*Ear symptoms*					
Yes	4 (3.51)	—	3 (18.75)	1 (2)	≤0.001
No	45 (39.47)	41 (85.42)	2 (12.5)	1 (2)	
Did not tell	65 (57.02)	7 (14.58)	11 (68.75)	48 (96)	
Total	114 (100)	48 (100)	16 (100)	50 (100)	
*Self-rated health condition*					
Very good	13 (11.4)	2 (4.17)	7 (43.75)	4 (8)	≤0.001
Good	56 (49.12)	24 (50)	7 (43.75)	25 (50)	
Average/fair	36 (31.58)	18 (37.5)	—	18 (36)	
Poor	6 (5.26)	4 (8.33)	—	2 (4)	
Did not tell	3 (2.63)	—	2 (12.5)	1 (2)	
Total	114 (100)	48 (100)	16 (100)	50 (100)	
*Mental health disorders*					
At risk	24 (21.05)	8 (16.67)	5 (31.25)	11 (22)	0.460
Not at risk	90 (78.95)	40 (83.33)	11 (68.75)	39 (78)	
Did not tell	—	—	—	—	
Total	114 (100)	48 (100)	16 (100)	50 (100)	
*Clinic/hospital visit in the last 1 year*					
Yes	62 (54.39)	27 (56.25)	3 (18.75)	32 (64)	0.006
No	52 (45.61)	21 (43.75)	13 (81.25)	18 (36)	
Did not tell	—	—	—	—	
Total	114 (100)	48 (100)	16 (100)	50 (100)	
*Smoking habit*					
Yes	50 (43.86)	23 (47.92)	5 (31.25)	22 (44)	0.617
No	63 (55.26)	25 (52.08)	10 (62.5)	28 (56)	
Did not tell	1 (0.9)	—	1 (6.25)	—	
Total	114 (100)	48 (100)	16 (100)	50 (100)	
*Alcohol usage*					
Yes	66 (57.89)	30 (62.5)	4 (25)	32 (64)	0.015
No	48 (42.11)	18 (37.5)	12 (75)	18 (36)	
Did not tell	—	—	—	—	
Total	114 (100)	48 (100)	16 (100)	50 (100)	
*Eating at the landfill site*					
Yes	77 (67.54)	33 (68.75)	11 (68.75)	33 (66)	0.954
No	37 (32.46)	15 (31.25)	5 (31.25)	17 (34)	
Did not tell	—	—	—	—	
Total	114 (100)	48 (100)	16 (100)	50 (100)	
*Landfill site safety*					
Yes	79 (69.30)	39 (81.25)	3 (18.75)	37 (74)	≤0.001
No	35 (30.70)	9 (18.75)	13 (81.25)	13 (26)	
Did not tell	—	—	—	—	
Total	114 (100)	48 (100)	16 (100)	50 (100)	
*Infectious diseases*					
Yes	2 (1.75)	—	—	2 (4)	0.261
No	108 (94.74)	48 (100)	15 (93.75)	45 (90)	
Did not tell	4 (3.51)	—	1 (6.25)	3 (6)	
Total	114 (100)	48 (100)	16 (100)	50 (100)	
*Chronic diseases*					
Yes	1 (0.88)	—	—	1 (2)	0.518
No	111 (97.37)	48 (100)	16 (100)	47 (94)	
Did not tell	2 (1.75)	—	—	2 (4)	
Total	114 (100)	48 (100)	16 (100)	50 (100)	

**Table 3:** The association between landfill exposure and mental health disorders.

Factor	*B*	Crude OR_a_ (95% CI)	Adjusted OR_b_ (95% CI)	*P* value
Landfill exposure	0.566	1.633 (1.032, 3.288)	1.733 (1.069, 2.755)	0.041^*∗*^
Mental health disorder	1.220	2.631 (1.164, 4.877)	1.540 (1.252, 5.664)	0.003^*∗*^

^a^Simple logistic regression. ^b^Multiple logistic regression. ^*∗*^Statistically significant (0.05).

**Table 4:** Association between landfill exposure and number of days worked.

Factor	*b*	Crude OR_a_ (95% CI)	Adjusted OR_b_ (95% CI)	*P* value
Landfill exposure	0.488	1.301 (1.021, 3.042)	1.449 (1.072, 2.628)	0.034^*∗*^
Number of days worked	1.261	2.352 (1.125, 4.533)	2.126 (1.281, 4.221)	0.005^*∗*^

^a^Simple logistic regression. ^b^Multiple logistic regression. ^*∗*^Statistically significant (0.05).

**Table 5:** Association between landfill exposure, age, and infectious and chronic diseases.

Factor	*b*	Crude OR_a_ (95% CI)	Adjusted OR_b_ (95% CI)	*P* value
Landfill exposure	0.237	1.255 (1.094, 3.292)	1.355 (1.102, 2.121)	0.025^*∗*^
Age	1.109	2.103 (1.824, 4.048)	2.217 (1.108, 3.311)	0.007^*∗*^
Infectious diseases	0.164	1.642 (1.0008, 1.039)	2.081 (1.349, 2.109)	0.021^*∗*^
Chronic diseases	0.184	1.035 (1.081, 1.042)	2.136 (1.406, 2.254)	0.004^*∗*^

^a^Simple logistic regression. ^b^Multiple logistic regression. ^*∗*^Statistically significant (0.05).

**Table 6:** Observation of occupational and hazardous exposures at the landfill sites.

	Observations	Landfill A	Landfill B	Landfill C
Common hazardous substance	Waste pickers were exposed to different hazardous chemical substances dumped at the landfill sites	The landfill management has a specially protected area for the dumping of hazardous waste, but some still find their way to the main landfill sites. Most of the waste pickers only had surgical masks (which were believed to be because of the COVID-19 protocol) as against proper N95 masks. These masks cannot protect the waste pickers against the breathing in of toxic fumes. No eye goggle was worn by the waste pickers.	The control measures observed were not adequate in protecting the waste pickers against inhaling toxic substances. Most of the waste pickers did not wear a suitable mask. A few wear surgical masks, and others use fabrics as masks. No eye goggle was worn by the waste pickers.	Waste pickers were mainly on surgical masks and fabrics as PPE. No eye goggle was worn by the waste pickers.

Dust	Building rubbles are used in stabilising the roads, thus creating/emitting dust which the waste pickers are exposed to. Other sources of the airborne dust observed in the landfill sites include dust liberated from waste materials by compactor and dump trucks. Dust may comprise organic matters which may lead to dermatological and respiratory symptoms.	Most of the waste pickers did not wear protective dust masks to protect themselves from inhaling dust while they haul out recyclable waste materials. Water tanker occasionally is used to wet the soil.	Water tanker frequently used in wetting the soil, but waste pickers were still exposed as they do not have the proper PPE	Water tanker occasionally used to stabilise the soil. Waste pickers are not free from exposure as most are not properly kitted.

## Data Availability

The data used to support the findings of the study are available from the corresponding author upon request.

## Supplementary Materials

Supplementary Materials Questionnaire survey.
