# Supplementary material for: Occupational Health Risk of Waste Pickers: A Case Study of Northern Region of South Africa
Source: J Environ Public Health. 2021 Aug 30;2021:5530064. doi: 10.1155/2021/5530064 (PMC8424242; doi:10.1155/2021/5530064)
Supplement: Supplementary Materials — Questionnaire survey. [file 5530064.f1.docx]

**SECTION A: Demographic and socio-economic characteristics information**

**1. Gender**

| Male | 1 |  |
| --- | --- | --- |
| Female | 2 |  |

**2. Age in years**

| Less than 11 | 1 |  |
| --- | --- | --- |
| 12-20 | 2 |  |
| 21-30 | 3 |  |
| 31-40 | 4 |  |
| 41-50 | 5 |  |
| 51 and above | 6 |  |

**3. Marital status**

| Single | 1 |  |
| --- | --- | --- |
| Married | 2 |  |
| Divorced | 3 |  |
| Widowed/Widower | 4 |  |

**4. What is your highest level of education?**

| No formal education | 1 |  |
| --- | --- | --- |
| Primary | 2 |  |
| Secondary | 3 |  |
| Basic degree | 4 |  |
| Honours degree and above | 5 |  |

**5. What are your occupation details?**

| Employed | 1 |  |
| --- | --- | --- |
| Self employed | 2 |  |
| Unemployed | 3 |  |

**6. Years of employment**

| 1-3 | 1 |  |
| --- | --- | --- |
| 4-5 | 2 |  |
| 6 | 3 |  |

**7. What is your monthly income?**

| R0-500 | 1 |  |
| --- | --- | --- |
| R501-1000 | 2 |  |
| R1001-1500 | 3 |  |
| R1501-3500 | 4 |  |
| R3501-5000 | 5 |  |
| R5001 and above | 6 |  |

**8. How many day a week do you work?**

| 1 day | 1 |  |
| --- | --- | --- |
| 2 days | 2 |  |
| 3 days | 3 |  |
| 4 days | 4 |  |
| 5 days | 5 |  |
| 6 days | 6 |  |
| 7 days | 7 |  |

**9. Is waste picking your full-time job?**

| Yes | 1 |  |
| --- | --- | --- |
| No | 2 |  |

**10. If no, what other jobs do you?**

|  | 1 |  |
| --- | --- | --- |
|  | 2 |  |
|  | 3 |  |
|  | 4 |  |
|  | 5 |  |
|  | 6 |  |

**11. Number of dependants?**

| 1 | 1 |
| --- | --- |
| 2 | 2 |
| 3 | 3 |
| 4 | 4 |
| 5 and above | 5 |

**B. Waste collection**

| General household waste | 1 |  |
| --- | --- | --- |
| Plastics | 2 |  |
| Cardboard | 3 |  |
| Metals | 4 |  |
| Bottles | 5 |  |
| Others (Specify) | 6 |  |

**12. Type of waste sorted**

| Yes | 1 |  |
| --- | --- | --- |
| No | 2 |  |

**13. Do you normally use Personal Protective Equipment?**

| Gloves | 1 |  |
| --- | --- | --- |
| Nose mask | 2 |  |
| Eye goggle | 3 |  |
| Boot | 4 |  |
| Coverall | 5 |  |
| Closed shoes | 6 |  |
| Others (Specify) | 7 |  |

**14. If yes, which of the Personal Protective Equipment do you use?**

**15. Does waste picking requires lifting heavy objects**

| Yes | 1 |  |
| --- | --- | --- |
| No | 2 |  |

**B. Health symptoms in the last 1 year**

**16. Association with any of the following**

| Headache | 1 |  |
| --- | --- | --- |
| Weakness | 2 |  |
| Flu | 3 |  |
| Loss of appetite | 4 |  |
| Others (Specify) | 5 |  |

**17. Association with any of the following respiratory symptoms?**

| Cough | 1 |  |
| --- | --- | --- |
| Phlegm | 2 |  |
| Wheezing | 3 |  |
| Asphyxiate | 4 |  |
| Chest pain | 5 |  |
| Sore throat | 6 |  |
| Others (Specify) | 7 |  |

**18. Association with any of the following gastrointestinal symptoms?**

| Nausea & vomiting | 1 |  |
| --- | --- | --- |
| Diarrhoea | 2 |  |
| Stomach ache | 3 |  |
| Bloody stool | 4 |  |
| Constipation | 5 |  |
| Heartburn | 6 |  |
| Abdominal pain | 7 |  |
| Dysentery | 8 |  |
| Others (Specify) | 9 |  |

**19. Dermatological (Skin) symptoms**

| Itchy | 1 |  |
| --- | --- | --- |
| Rash | 2 |  |
| Peeling skin | 3 |  |
| Dry, cracked skin | 4 |  |
| Discoloured patches of skin | 4 |  |
| Hot irritated skin | 6 |  |
| Others (Specify) | 7 |  |

**20. Musculoskeletal symptoms**

| General aches and body pains | 1 |  |
| --- | --- | --- |
| Low back pain | 2 |  |
| Elbow/wrist pain | 3 |  |
| Problem of body posture | 4 |  |
| Excessive stretching muscle | 5 |  |
| Bend, sprain and swollen of body | 6 |  |
| Others (Specify) | 7 |  |

**21. Eye symptoms**

| Eye irritation | 1 |  |
| --- | --- | --- |
| Blurry vision | 2 |  |
| Eye infection | 3 |  |
| Night blindness | 4 |  |
| Others (Specify) | 5 |  |

**22. Ear symptoms**

| Hearing problem | 1 |  |
| --- | --- | --- |
| Ear pain | 2 |  |
| Ear infection | 3 |  |
| Loss of hearing | 4 |  |
| Others (Specify) | 5 |  |

**C. Self-rated health**

**23. What is your health condition?**

| Very good | 1 |  |
| --- | --- | --- |
| Good | 2 |  |
| Average/fair | 3 |  |
| Poor | 4 |  |
| Others (Specify) | 5 |  |

**24. How would you rate your mental health?**

| At risk | 1 |  |
| --- | --- | --- |
| Not at risk | 2 |  |

**25. Have you visited the clinic/hospital in the last 1 year?**

| Yes | 1 |  |
| --- | --- | --- |
| No | 2 |  |

| Yes | 1 |  |
| --- | --- | --- |
| No | 2 |  |

**26. Do you smoke?**

**27. Alcohol usage**

| Yes | 1 |  |
| --- | --- | --- |
| **No** | **2** |  |

**28. Landfill site eating**

| Yes | 1 |  |
| --- | --- | --- |
| No | 2 |  |

| Yes | 1 |  |
| --- | --- | --- |
| No | 2 |  |

**29. Landfill site safety**

**30. Infectious diseases**

| Yes | 1 |  |
| --- | --- | --- |
| No | 2 |  |

**31. Chronic diseases**

| Yes | 1 |  |
| --- | --- | --- |
| No | 2 |  |

**Thank you for participating in this survey!**

WHO 20 Self Reporting Questionnaire

| 1 | Do you often have headaches? | Yes | No |
| --- | --- | --- | --- |
| 2 | Is your appetite poor? | Yes | No |
| 3 | Do you sleep badly? | Yes | No |
| 4 | Are you easily frightened | Yes | No |
| 5 | Do your hand shake? | Yes | No |
| 6 | Do you feel nervous, tense or worried? | Yes | No |
| 7 | Is your digestion poor? | Yes | No |
| 8 | Do you have trouble thinking clearly? | Yes | No |
| 9 | Do you feel unhappy? | Yes | No |
| 10 | Do you cry more than usual? | Yes | No |
| 11 | Do you find it difficult to enjoy your daily activities | Yes | No |
| 12 | Do you find it difficult to make decisions? | Yes | No |
| 13 | Is your work suffering? | Yes | No |
| 14 | Are you unable to play a useful part of life? | Yes | No |
| 15 | Have you lost interest in things? | Yes | No |
| 16 | Do you feel that you are a worthless person? | Yes | No |
| 17 | Has the thought of ending your life been on your mind? | Yes | No |
| 18 | Do you feel tired all the time? | Yes | No |
| 19 | Do you have uncomfortable feelings in your stomach? | Yes | No |
| 20 | Are you easily tired? | Yes | No |
